# Yet another reason we need to tackle socioeconomic inequalities in smoking

**DOI:** 10.1016/j.lanepe.2022.100520

**Published:** 2022-09-29

**Authors:** Michael J. Green

**Affiliations:** MRC/CSO Social & Public Health Science Unit, University of Glasgow, United Kingdom

Smoking is a major contributor to socioeconomic inequalities for a wide range of health conditions. A link between smoking and dementia has been uncertain but prior evidence indicates socioeconomic inequalities in both smoking and dementia, so Raggi et al.^1^ set out to quantify whether and how much socioeconomic inequalities in smoking contribute to socioeconomic inequalities in dementia. With data on smoking and occupational grade from the UK Whitehall II cohort study and approximately 30 years of follow up, they provide robust observational estimates, supported by a commendable range of sensitivity analyses, indicating that smoking increases risk of later life dementia and that mid-life smoking status and smoking history can contribute to socioeconomic inequalities in dementia. Notably, strong contributions to inequalities in dementia were found for current smoking in mid-life, with little evidence for increased risk among smokers who had quit earlier. Thus, considering the 15-20 year pre-clinical phase of dementia, smoking cessation should ideally be recommended as early as possible and long before dementia symptoms appear, which is hardly a new recommendation. Contributions of smoking to socioeconomic inequalities in dementia were estimated as fairly modest (at 16%), but the magnitude is probably less important. Dementia belongs among the many health conditions for which smoking contributes to socioeconomic inequalities. The findings add to an extensive evidence base already urging us to consider what policies or interventions might be most effective, not just to reduce smoking rates, but to alleviate socioeconomic inequalities in smoking.

Inequalities in smoking can arise from a combination of inequalities in smoking uptake during youth and young adulthood and inequalities in smoking cessation among adults.^2^ Importantly then, indicators of socioeconomic position such as education, occupation or income may be associated with distinct causal mechanisms and be more or less important at different life stages.^3^^,^^4^ Education usually occurs when smoking behaviours are being established and can represent problem-solving skills that help with behaviour change,^5^ while occupational measures are more indicative of day-to-day physical and social environments during adulthood when smoking habits are already established. Consider that Raggi et al.^1^ estimated effects for a measure of occupation with adjustment for education. Excluding the contribution of education will underestimate the overall socioeconomic inequality in both smoking and dementia. Nevertheless, adjustment is appropriate for estimating the specific effect of occupational grade because education confounds these relationships, i.e. it is a potential determinant of occupational grade, smoking and dementia risk. Thus, the study makes an informative contribution by indicating that a person's adult, daily working and social environment and not just their educational background, can influence risk for smoking and for dementia.

When implementing policies or interventions aimed at reducing smoking rates, a more consistent focus on socioeconomic inequalities would also be beneficial. For example, a recent adult-focused review of population-level measures found almost half of the 68 included studies had not been designed to assess intervention impact by socioeconomic position, and many had sub-optimal methodological characteristics for assessing this.^6^ Similar findings emerged in a review focused on adolescents.^7^ Price increases and targeted cessation seem most clearly evidenced for alleviating socioeconomic inequalities in smoking, but a great many other potential policies or interventions have mixed or unclear evidence.^6^^,^^7^ For example, we found that UK implementation of smoke-free legislation and a change in the legal age for cigarette purchase from 16 to 18 had been associated with a reduction in socioeconomic inequalities in smoking uptake among youth (aged 11–15),^8^ but the change was not immediately apparent, accumulating gradually over several years, and the two policies had been implemented so closely together that we could not distinguish whether either or both together were responsible. There is much still to learn about how the equity impact of interventions depends on factors such as the mode, scale and length of their implementation, and how different interventions interact with each other.^6^

Other open questions also remain. While this paper presents strong evidence that inequalities in smoking by occupational grade contribute to occupational grade inequalities in dementia,^1^ the role of other socioeconomic factors such as education is less clear. Since the Whitehall II cohort only sampled from a from a select working population and excluded the unemployed, we also know little about how smoking contributes to inequalities in dementia between those who do and do not work. Further research that carefully distinguishes differing socioeconomic measures, while acknowledging the causal links between them,^4^ could give useful insights to help focus interventions and policy more effectively on particular socioeconomic characteristics or stages of the life course. Furthermore, the emergence of e-cigarettes, now often used as smoking cessation aids,^9^ presents new questions about how these devices and differing regulatory approaches to them^10^ may impact on inequalities in smoking. Additionally, as Raggi et al.^1^ point out, smoking is still prevalent in low to middle income countries where dementia is on the rise, so understanding how equity impacts of policies and interventions differ across social contexts is vital.^6^ Research on such issues, that improves our understanding of how to alleviate socioeconomic inequalities in smoking could produce large dividends in alleviating socioeconomic inequalities, not just for dementia, but for many health conditions.

## Contributor

MJG wrote the manuscript.

## Declaration of interests

MJG received funding from the UK Medical Research Council (MC_UU_00022/2) and Scotland's Chief Scientist Office (SPHSU17). Funders had no role in preparation or submission of the manuscript.

## References

[1] Raggi M, Dugravot A, Valeri L (2022). Contribution of smoking towards the association between socioeconomic position and dementia: 32-year follow-up of the Whitehall II prospective cohort study. Lancet Regional Health Europe.

[2] Maralani V. (2013). Educational inequalities in smoking: The role of initiation versus quitting. Soc Sci Med.

[3] Galobardes B, Shaw M, Lawlor DA, Lynch JW, Davey Smith G (2006). Indicators of socioeconomic position (part 1). J Epidemiol Community Health.

[4] Green MJ, Popham F. (2019). Interpreting mutual adjustment for multiple indicators of socioeconomic position without committing mutual adjustment fallacies. BMC Public Health.

[5] Mirowsky J, Ross CE. (1998). Education, Personal Control, Lifestyle and Health. Res Aging.

[6] Smith CE, Hill SE, Amos A. (2021). Impact of population tobacco control interventions on socioeconomic inequalities in smoking: a systematic review and appraisal of future research directions. Tob Control.

[7] Brown T, Platt S, Amos A. (2014). Equity impact of interventions and policies to reduce smoking in youth: systematic review. Tob Control.

[8] Anyanwu PE, Craig P, Katikireddi SV, Green MJ. (2020). Impact of UK tobacco control policies on inequalities in youth smoking uptake: a natural experiment study. Nicotin Tob Res.

[9] Hartmann-Boyce J, McRobbie H, Lindson N (2020). Electronic cigarettes for smoking cessation. Cochrane Database Syst Rev.

[10] Smith MJ, Baxter AJ, Skivington K, McCann M, Hilton S, Katikireddi SV. (2021). Examining the sources of evidence in e-cigarette policy recommendations: A citation network analysis of international public health recommendations. PLoS One.

